# Circular RNAs in Cardiovascular Disease: Mechanisms, Biomarkers, and Therapeutic Frontiers

**DOI:** 10.3390/biom15101455

**Published:** 2025-10-15

**Authors:** Rudaynah Alali, Mohammed Almansori, Chittibabu Vatte, Mohammed S. Akhtar, Seba S. Abduljabbar, Hassan Al-Matroud, Mohammed J. Alnuwaysir, Hasan A. Radhi, Brendan Keating, Alawi Habara, Amein K. Al-Ali

**Affiliations:** 1 Department of Internal Medicine, King Fahad Hospital of the University, Al Khobar 34445, Saudi Arabia; raali@iau.edu.sa (R.A.); mmansori@iau.edu.sa (M.A.); alnowaysir@gmail.com (M.J.A.); hasan90radhi@gmail.com (H.A.R.); 2 Imam Abdulrahman Bin Faisal University, Dammam 31441, Saudi Arabia; 3 Department of Clinical Biochemistry, College of Medicine, Imam Abdulrahman Bin Faisal University, Dammam 31441, Saudi Arabia; cbvatte@iau.edu.sa (C.V.); makhtar@iau.edu.sa (M.S.A.); ahhabara@iau.edu.sa (A.H.); 4 College of Medicine, Imam Abdulrahman Bin Faisal University, Dammam 31441, Saudi Arabia; 2240004149@iau.edu.sa (S.S.A.); 2230005286@iau.edu.sa (H.A.-M.); 5 Department of Surgery, NYU Grossman School of Medicine, New York, NY 10016, USA; brendan.keating@nyulangone.org

**Keywords:** circular RNA, cardiovascular disease, atherosclerosis, coronary artery disease

## Abstract

Circular RNAs (circRNAs) have emerged as crucial cardiovascular regulators through gene expression modulation, microRNA sponging, and protein interactions. Their covalently closed structure confers exceptional stability, making them detectable in blood and tissues as potential biomarkers. This review explores current research examining circRNAs across cardiovascular diseases, including atherosclerosis, myocardial infarction, and heart failure. We highlight the control that circRNA exerts over endothelial function, smooth muscle switching, inflammatory recruitment, and cardiomyocyte survival. Key findings distinguish frequently disease-promoting circRNAs (circANRIL, circHIPK3) from context-dependent regulators (circFOXO3). Compartment-specific controllers include endothelial stabilizers (circGNAQ), smooth muscle modulators (circLRP6, circROBO2), and macrophage regulators (circZNF609), functioning as tunable rheostats across vascular compartments. Overall, the literature suggests that circRNAs represent promising tools in two translational avenues: (i) blood-based multimarker panels for precision diagnosis and (ii) targeted modulation of pathogenic circuits. Clinical translation will require precise cell-type targeting, efficient delivery to cardiovascular tissues, and rigorous mitigation of off-target effects.

## 1. Introduction

Circular RNAs (circRNAs) were initially reported in the 1970s and were initially dismissed as splicing artifacts [1,2,3,4,5]. Evolutionary conservation is a key translational filter for circRNA findings from animal models to humans. Because many circRNAs and their back-splice junctions are weakly conserved beyond syntenic exons, mouse results should be prioritized for translation when the human ortholog shows conserved junctions or sequence/structural features, heart tissue expression, and preserved interaction partners (miRNA/protein). Cross-species mapping and ortholog confirmation via RNase R-resistant, junction-spanning qPCR in human samples can lower the risk of species artifacts.

**Figure 1:**
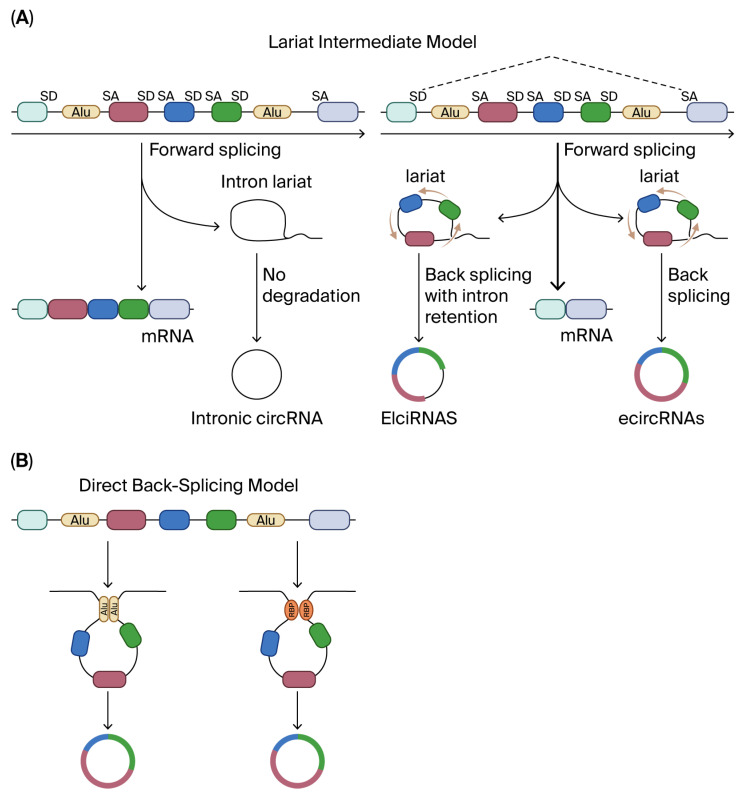
Mechanism for forming circRNA. (**A**) In the lariat intermediate model, linear RNA is produced first after canonical splicing, leaving a lariat that contains introns, and in other instances, the lariat will contain introns and the skipped exons. That lariat will form the circRNA. (**B**) In the direct back-splicing model, circRNA is produced first. This is facilitated by intronic complementary sequences (ICSs) that flank the circRNA sequence. The ICSs must be in two different intronic regions to create the loop that flanks the circRNA sequence. Alu elements are types of ICSs found in the human genome. Alu elements can form RNA duplexes, which promote interactions among other genetic elements and proteins that affect gene regulation. SD, splice donor; SA, splice acceptor; Alu, a type of short interspersed nuclear element that is about 300 base pairs long and is named after the restriction endonuclease Alul.

Refined bioinformatic pipelines combined with throughput RNA-seq have shown that circRNAs are numerous, conserved across many species, and generated via the back-splicing of precursor mRNAs [1,2,3,4,5] (Figure 1). This evidence suggests that this circRNA potentially contributes to miRNA sequestration; however, abundance, localization, and binding-site stoichiometry limit strong causal inference.

We qualify mechanistic claims by explicitly considering biological plausibility, including circRNA abundance relative to targets, subcellular localization, and stoichiometric constraints. Functional relevance typically requires high circRNA copy numbers, cytoplasmic localization, and an aggregate number of accessible miRNA response elements high enough to impact the effective miRNA pool; when these criteria are not met, alternative mechanisms may better explain observations.

**Figure 2:**
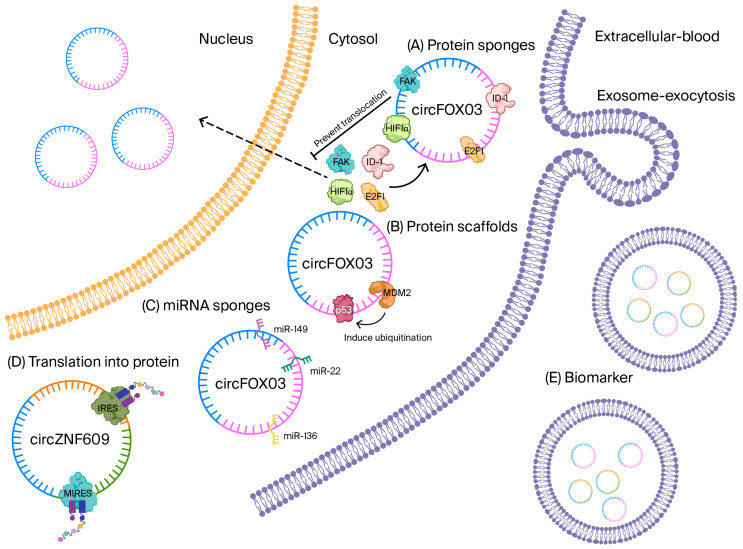
CircRNA mechanism. CircRNA interacts with proteins to perform various functions. (**A**) circFOXO3 contains multiple binding motifs for protein binding, such as ID-1, FAK, E2F1, and HIF1α, which leads to the sponging (sequestering) of these proteins, preventing their translocation to the nucleus. (**B**) Additionally, circFOXO3 has binding motifs for p53 and MDM2, but the function here is to induce the ubiquitination of p53, which is facilitated by interaction with MDM2. (**C**) circRNA can function as miRNA sponges, preventing their action. circFOXO3 has miRNA response elements (MREs), which are binding motif sequences for miR-149, miR-22, and miR-136. All these functions can be performed by circFOXO3, showing that these circRNA are versatile in gene regulation. (**D**) circRNA can translate into protein. circZNF609 has two specific sequences, internal ribosome entry sites (IRESs) and m6A-induced ribosome engagement sites (MIRESs), which can initiate translation in a eukaryote, and it is independent of the 5′cap structure and 3′ poly A tail. (**E**) circRNA can leave the cell in an exosome and enter the blood, where they can function as a biomarker for different disease conditions, such as CVD.

Looped topology confirms that circRNAs show an aversion to exonucleases, which enables them to exhibit extraordinary stability in both biofluids and tissues. This makes them very strong biomarker candidates. The influence of circRNAs is seen at multiple levels of gene regulation. The most frequently studied example is microRNA (miRNA) sequestration, where circRNAs, including ciRS-7, sponge miR-7 and activate broad mRNA networks by sheltering many miRNA response elements [6,7,8,9,10] (Figure 2).

CircRNA dysregulation is unlikely to be static; rather, expression likely follows disease trajectories with stage-, tissue-, and treatment-dependent shifts [11]. To enhance translational value, circRNA biomarkers should be evaluated longitudinally (serial sampling) with prespecified early versus late/acute versus chronic strata and reported with regard to their temporal discrimination and calibration, the modification of their effects via therapy, and their incremental prognostic value across stage-specific clinical models. For therapeutic targeting, candidates should demonstrate consistent directional change across time and reversibility with effective treatment, or stage-restricted activity that guides the timing of interventions.

Some circRNAs directly interact with epigenetic remodelers or transcription factors to control RNA-polymerase II activity and chromatin architecture [11,12,13].

A third layer has emerged with the discovery of internal ribosome entry sites (IRESs) and N^6^-methyladenosine (m^6^A)-mediated ribosome engagement sites that allow for cap-independent translation; dozens of circRNA-encoded peptides with discrete biological functions have now been cataloged [14,15,16,17]. Finally, certain circRNAs serve as protein scaffolds or decoys, orchestrating multi-protein complexes, for example, circFOXO3 tethers p53 and MDM2, accelerating p53 ubiquitination while stabilizing the pro-apoptotic transcription factor FOXO3 [18]. Table 1 summarizes the mechanistic role of circRNA.

Beyond basic cell biology, circRNAs are increasingly implicated in human disease. Cardiovascular diseases (CVDs) remain the leading cause of global mortality, with diagnostic and prognostic gaps persisting, despite advances in troponins, natriuretic peptides, and advanced imaging [19,20]. Recent RNA-seq surveys of human myocardium, atherosclerotic plaques, and circulating blood cells have uncovered hundreds of dysregulated circRNAs across coronary artery disease (CAD), myocardial infarction (MI), cardiac hypertrophy, and heart failure [21,22,23,24]. Functional follow-up studies highlight diverse roles: circANRIL induces nucleolar stress in vascular smooth muscle cells, thereby limiting plaque stability [24], and circMYO9A promotes cardiomyocyte hypertrophy by sponging miR-26b-5p [23]. Nonetheless, most investigations have been limited to single centers and small cohorts, underscoring the need for systematic synthesis.

In the sections that follow, we will (i) catalog circRNAs frequently linked to cardiac hypertrophy, atherosclerosis, and coronary artery disease and (ii) evaluate their translational readiness as biomarkers or therapeutic targets. By integrating mechanistic depth into clinical evidence, this review will suggest a roadmap for circRNA-based precision cardiology. For biomarker studies, we report the key details to support appraisal and generalizability: patient characteristics (age, sex, ancestry, stage/severity, comorbidities, treatment, inclusion/exclusion), control definitions (healthy vs. disease, matching, source), and analytical methods (pre-analytics, platform/normalization, QC, blinding, batch correction, prespecified thresholds), plus sample sizes, missing-data handling, and internal/external validation.

## 2. circRNAs Involved in Cardiac Hypertrophy

Cardiac hypertrophy signifies the initial compensatory response to chronic hemodynamic overload. A narrowed aortic valve results in high blood pressure, which then increases pressure on the heart; this causes heart muscle cells to increase in size to sustain normal pumping function [25,26,27,28,29,30]. This adaptive phase is energy-efficient, limiting oxygen consumption for a given afterload. Continued stress on the heart leads to the muscles becoming unhealthy. This results in the formation of scar tissue, abnormal gene activity, damage to energy-producing structures, and a reduction in blood supply. Ultimately, in a relaxed state, the heart will fail and pumping will decrease, which in turn leads to dangerous heart rhythms, which can end in sudden death [29,30,31,32]. The growth of the heart muscle is controlled by several key signaling pathways, and blocking only one of the pathways will only partially slow the progression of heart disease [31,32].

In the past five years, non-coding RNAs, particularly circRNAs, have emerged as essential regulators of this phenotypic switch. The use of advanced gene analysis on enlarged mouse hearts has found that a specific RNA molecule, circMYO9A, makes heart muscle cells grow larger. It works by blocking protective molecules and allowing a growth-promoting gene (Gata4) to become more active [23]. Subsequent gain- and loss-of-function studies demonstrated that circNfix modulates the Hippo–YAP pathway to govern post-MI cardiac regeneration, while circSlc8a1 antagonizes miR-133a to exacerbate pressure-overload hypertrophy in vivo [33,34].

These results show that circular RNAs act as key controllers in how heart cells respond to stress and handle calcium. This suggests that we could develop new treatments using specially designed molecules or gene therapy to target these RNA interactions. There is a need to continue studying how circular RNAs interact with other molecules, where they are located in cells, and whether they synthesize proteins. This research is essential in translating our scientific discoveries into new treatments for unhealthy heart enlargement.

### 2.1. CircHRCR, Guardian of the Stressed Myocardium

CircHRCR plays a significant role in cardiac biology, particularly in regulating cardiac hypertrophy and heart failure [30]. CircHRCR achieves its protective effects through several mechanisms. Firstly, circHRCR acts as a molecular sponge for miR-223. By sponging miR-223, circHRCR indirectly upregulates the expression of apoptosis repressors with the caspase recruitment domain (ARC), an anti-apoptotic protein, thus protecting cardiomyocytes from apoptosis and subsequent heart failure [30]. Additionally, the sponging activity of circHRCR against miR-223 plays a role in regulating cardiac hypertrophy by preventing the downregulation of anti-hypertrophic genes, such as *FBXW7* and *RASA1*, attenuating the pathological growth of cardiomyocytes, and acting as a therapeutic target for cardiac hypertrophy [30,35]. circHRCR protects the heart by mitigating the effects of stress and the pathological stimuli leading to cardiac hypertrophy and heart failure. Its ability to interact with miR-223 and influence the expression of key proteins involved in cell survival and growth indicates its potential as a target for therapeutic interventions in cardiovascular diseases.

### 2.2. CircHIPK3, Driver of Maladaptive Hypertrophy

CircHIPK3 is a circular RNA that has been found to play a detrimental role in cardiac hypertrophy. It exacerbates cardiac hypertrophy and dysfunction by acting as a molecular sponge for miR-185-3p [36]. By sequestering miR-185-3p, circHIPK3 prevents this miRNA from downregulating its target genes involved in promoting hypertrophic responses, such as *CaMKIIδ* and *NCX1* [36], contributing to the progression of cardiac hypertrophy and associated cardiac dysfunction. Thus, circHIPK3 can be used as a biomarker for cardiac hypertrophy and the modulation of the level of circHIPK3, which can attenuate cardiac hypertrophy, making it a potential target for therapeutic interventions to mitigate hypertrophic heart disease.

### 2.3. CircYAP1, Hippo-Pathway Satellite That Guards Against Fibrosis and Hypertrophy

Derived from the *YAP1* gene, circYAP1 is crucial in reducing cardiac fibrosis and influencing cardiac hypertrophy through its unique protein interactions [37]. A key regulator in the Hippo signaling pathway, YAP1 significantly affects cardiac hypertrophy by promoting cardiomyocyte proliferation and survival [34]. YAP1 moves to the nucleus upon activation and interacts with transcription factors, such as TEADs, to stimulate gene expression in cell growth and survival [37]. YAP1 also inhibits apoptosis, thus supporting cardiomyocyte survival under stress conditions and responding to mechanical stress to facilitate adaptive growth responses. Notably, no evidence indicates that circYAP1 binds directly to YAP1 [37]; instead, circYAP1 binds to Actin Gamma-1 (ACTG) and Tropomyosin 4 (TPM4), which are essential in maintaining cell structure and function [34]. By interacting with these proteins, circYAP1 helps regulate the structural integrity and functionality of cardiomyocytes, enhancing the protective effects of YAP1. Together, circYAP1 and YAP1 mitigate cardiac hypertrophy and fibrosis, highlighting their potential as therapeutic targets in the management of hypertrophic and fibrotic heart diseases.

### 2.4. CircSLC8A1, Calcium-Handling Hub That Fuels Pathological Growth

circSLC8A1 originates from the *SLC8A1* gene (also known as *NCX1*). Gene analysis of mouse heart cells that had been treated with a growth hormone and hearts from stressed mice revealed that one of the circular RNAs that increased the most during heart enlargement was circSLC8A1 [34,38]. CircSLC8A1 functions by sponging miR-133a-3p, which is a microRNA that usually averts heart growth and remodeling. However, when miR-133a-3p is not present, some genes that encourage heart growth become more active, including the following:

• SERCA2a (*Atp2a2*), improving sarcoplasmic reticulum Ca^2+^ re-uptake and enabling stronger contractions;
• CTGF, a profibrotic matricellular protein;
• RhoA, which remodels the actin cytoskeleton and augments cell size;
• *Ccnd2*, a G1/S cyclin that extends the cardiomyocyte growth window.

Gain-of-function experiments have confirmed that circSLC8A1 overexpression enlarges cultured cardiomyocytes, heightens fetal gene markers (Nppa, Nppb), and worsens left-ventricular wall thickening and fibrosis in vivo, whereas targeted siRNA knockdown reverses these indices and normalizes chamber compliance [34,38]. Collectively, these data position circSLC8A1 at the crossroads of Ca^2+^ handling, cytoskeletal dynamics, and extracellular matrix turnover, making it an attractive target in antisense or CRISPR-Cas13 therapies aimed at halting maladaptive hypertrophy.

### 2.5. CircMYO9A, an NF-κB-Responsive Amplifier of the GATA4 Axis

**Figure 3:**
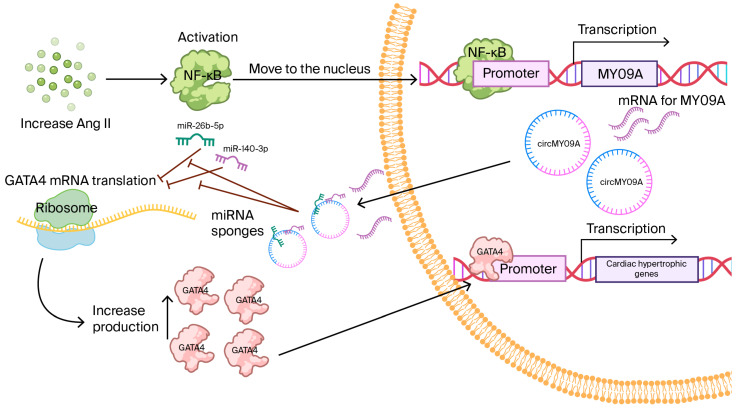
NF-κB-circMYO9A-miRNA-GATA4 Pathway in Cardiac Hypertrophy. This Figure represents the regulatory role of circMYO9A in cardiac hypertrophy. Angiotensin II (Ang-II) activates NF-κB signaling, leading to the upregulation of circMYO9A. Then, circMYO9A functions as a sponge for miR-26b-5p and miR-140-3p, preventing them from binding to the 3′-UTR of Gata4 mRNA. This relieves the inhibitory effect of these microRNAs, resulting in increased GATA4 protein expression. Elevated GATA4 drives the expression of hypertrophic genes, thereby promoting pathological cardiac hypertrophy.

Studies in immature mouse heart cells treated with growth hormone first identified circMYO9A, derived from the Myo9a gene [26,39]. During early cardiac hypertrophy, circMYO9A is strongly upregulated. Experimental assays have shown that circMYO9A contains binding sites for miR-26b-5p and miR-140-3p, two microRNAs that normally suppress the pro-hypertrophic transcription factor GATA4 [26,39]. When growth hormone is given or the aorta is artificially narrowed, NF-κB is activated and enhances Myo9a transcription, leading to higher levels of both linear RNA and circMYO9A [26,39] (Figure 3). The rise in circMYO9A sequesters protective microRNAs, allowing GATA4 to become more active and induce the re-expression of fetal genes in adult hearts. In mice with narrowed aortas, the overexpression of circMYO9A enlarged cardiomyocytes, increased fibrosis, and reduced cardiac function, while the silencing of circMYO9A improved relaxation and attenuated hypertrophy [26,39]. In addition, laboratory experiments suggest that circMYO9A can bind the RNA-binding protein QKI, indicating a potential role in regulating RNA stability. circMYO9A has been shown to associate with the RNA-binding protein QKI in binding assays (e.g., RIP/CLIP or pull-down); further studies are needed to determine the precise binding sites and the downstream consequences of splicing, stability, and/or subcellular localization in cardiomyocytes [26,39].

Collectively, these findings position circMYO9A as a nodal point linking inflammatory (NF-κB) cues with hypertrophic gene programs. The therapeutic silencing of circMYO9A, either with antisense oligonucleotides or CRISPR–Cas13, therefore represents an attractive strategy against maladaptive remodeling. However, CRISPR-Cas13 faces topology-specific hurdles with circRNAs: the lack of 5′/3′ ends and compact structures limit guide accessibility, making BSJ-spanning guides preferable, but their efficiency varies in vivo. Even after cleavage, back-splicing can regenerate the circle, so durable knockdown may require multiplexed guides and/or biogenesis blockade (e.g., disrupting intronic pairing or RBP sites). Off-target and collateral effects necessitate the design of high-specificity guides, the engineering of low-collateral Cas13 variants, and transcriptome-wide off-target screens. Success also depends on subcellular localization, cardiovascular cell-type delivery, and dosing that sustains on-target activity without innate immune activation.

### 2.6. CircWWP1 Mitigates β-Adrenergic-Driven Hypertrophy

By downregulating the expression of the atrial natriuretic factor (ANF) and miR-23a, circWWP1 also plays an inhibitory role in cardiac hypertrophy [40]. In mouse models of cardiac hypertrophy induced by isoproterenol hydrochloride, circWWP1 is significantly downregulated [40]. The overexpression of circWWP1 in cardiomyocytes decreased ANF and miR-23a levels, which are typically upregulated in hypertrophic hearts [40]. This finding suggests that circWWP1 exerts protective effects against cardiac hypertrophy by modulating the expression of these hypertrophic markers [40]. The interaction between circWWP1 and these downstream targets highlights its potential as a therapeutic target in the prevention or treatment of cardiac hypertrophy.

### 2.7. CircPAN3, an m6A-Sensitive Safeguard That Counteracts Isoproterenol Hypertrophy

In cardiac hypertrophy, multiple circRNAs converge on shared signaling axes, notably, PI3K–AKT, MAPK–ERK, and TGF-β/Smad, most commonly via miRNA-mediated ceRNA mechanisms and less frequently via protein scaffolding. To avoid redundancy, we will summarize these common cascades here and, in subsequent examples, highlight only circRNA-specific miRNA targets, cell types, and directionality.

circPAN3 attenuates cardiac hypertrophy through the miR-320-3p/HSP20 axis [38]. In both in vitro and in vivo models, circPAN3 expression was significantly reduced in response to isoproterenol (ISO)-induced cardiac hypertrophy [41]. The overexpression of circPAN3 in cardiomyocytes mitigates hypertrophy, reducing the cell surface area and levels of hypertrophic markers such as ANP, BNP, and β-MHC [41]. Mechanistically, circPAN3 acts as a sponge for miR-320-3p, thus preventing miR-320-3p from downregulating HSP20, a protein known to protect against cardiac hypertrophy [38]. This interaction highlights the importance of circPAN3 in maintaining cardiac health by regulating the miR-320-3p/HSP20 pathway. Additionally, circPAN3’s stability is influenced by N6-methyladenosine (m6A) modification, regulated by the m6A demethylase, *ALKBH5*, which is downregulated in hypertrophic conditions, leading to reduced circPAN3 levels [41]. These findings suggest that circPAN3 plays a protective role against cardiac hypertrophy by modulating key molecular pathways and could serve as a potential therapeutic target in the management of heart disease.

## 3. circRNAs in Atherosclerosis—Orchestrators of Plaque Formation and Vascular Inflammation

Atherosclerosis is a complex inflammatory disease that results from lipid accumulation, smooth muscle cell migration, endothelial dysfunction, and macrophage foam cell formation within the arterial walls [42,43,44]. High-throughput RNA-seq of human carotid plaques, coronary specimens, and circulating monocytes has revealed hundreds of dysregulated circular RNAs that correlate with plaque burden, stenosis severity, and adverse cardiovascular events [45,46].

Circular RNAs affect the behavior of smooth muscle cells, the response of immune cells, and the leakiness of blood vessels through related but differing molecular pathways [46]. circANRIL, circCDR1as, and circHIPK3 can be quantified in the blood and can be found in differing levels in patients with confirmed heart artery blockages compared to healthy individuals [46]. Their exonuclease resistance and cell type-specific back-splicing patterns make them particularly attractive as liquid biopsies for early atherosclerosis detection, risk stratification, and therapeutic monitoring [46].

Functionally, atherosclerosis-linked circRNAs converge on several core pathways: (i) endothelial nitric oxide synthase (eNOS) regulation and barrier function, (ii) smooth muscle cell contractile-to-synthetic phenotype transitions, (iii) macrophage M1/M2 polarization and foam cell formation, and (iv) extracellular matrix remodeling and plaque stability [46]. By modulating these processes through microRNA sponging, protein scaffolding, and in select cases peptide translation, circRNAs emerge as both drivers and brakes in atherogenesis. We will now outline the most frequently studied examples, which have been explored by various research groups; these are the circRNAs that show the highest potential for use in diagnosis or treatment.

### 3.1. CircANRIL Exhibits Context-Dependent Roles

Evidence indicates that interaction with PES1 can induce nucleolar stress and impair rRNA maturation, a mechanism linked to atheroprotection in human genetic studies, while other reports describe potentially detrimental effects under distinct isoform, tissue, or disease-stage conditions [46]. Pressure can limit the growth and activity of rapidly dividing muscle cells, which reduces the development of plaque in blood vessels. Therefore, circANRIL inhibits the excessive growth of cells within plaque that is developing [19,47]. However, the stress may weaken the protective covering of plaque by reducing repair cells if it occurs at the wrong time or is excessively strong. Additionally, the stress response can cause harmful molecules and changes in blood flow to damage the lining of the blood vessels, which may result in the rupture of plaques [19,47]. This duality explains why circANRIL has been described as both protective (limiting lesion expansion) and potentially detrimental (promoting senescence/apoptosis in cap-stabilizing compartments) depending on the cellular milieu and disease stage.

The contradictory reports of protective versus deleterious effects of circANRIL likely reflect interplay among the cell type, isoform heterogeneity, inflammatory milieu, and genetic background. In early or quiescent contexts, endothelium and contractile VSMCs exhibit circANRIL patterns consistent with restrained proliferation and enhanced autophagy; in advanced plaques, enrichment in synthetic-state VSMCs and activated macrophages—where TNF-α/IL-1β, oxidized LDL, and hypoxia are prominent—can rebalance ribosomal biogenesis and cell-cycle control toward maladaptive remodeling. At the locus level, 9p21 risk haplotypes and Polycomb-linked chromatin states may favor circularization and isoform usage, while splicing-factor availability influences back-splicing efficiency. Methodologically, bulk-tissue averaging, non-physiological overexpression, and incomplete isoform resolution can lead to apparent discrepancies. A rigorous resolution will require (i) isoform-specific perturbations at endogenous levels; (ii) cell type-resolved readouts in human plaques (single-cell and spatial); (iii) stratification by 9p21 genotype and inflammatory burden; and (iv) standardized analytic pipelines with RNase R controls and validated back-splice detection.

Emerging single-cell and spatial transcriptomic studies suggest that circANRIL exhibits marked context specificity across atherosclerotic plaques, with its expression varying according to the cell type, the lesion stage, and microenvironmental cues. In early lesions, circANRIL is more readily detected in endothelial cells and contractile vascular smooth muscle cells (VSMCs), where it aligns with anti-proliferative, pro-autophagic programs that limit neointimal expansion; in contrast, in advanced plaques, it is enriched in dedifferentiated, synthetic-state VSMCs and activated macrophages at the plaque shoulder, a niche characterized by a high level of inflammatory signaling, oxidized lipid burden, and disturbed shear stress. This spatial shift is consistent with reports that cytokines (TNF-α, IL-1β), oxidized LDL, and hypoxia-inducible pathways modulate circularization efficiency at the ANRIL locus (9p21) and influence isoform usage, potentially rebalancing circANRIL’s effects on ribosomal biogenesis and cell-cycle control. Moreover, allele-specific regulation at 9p21 and epigenetic remodeling (e.g., Polycomb activity) may further tune circANRIL abundance across individuals and plaque regions. Together, these data support a model in which circANRIL acts as a microenvironment-responsive rheostat: in homeostatic or early-lesion contexts, higher circANRIL restrains VSMC proliferation and fosters resolutive phenotypes, whereas in inflamed, lipid-rich microdomains of advanced plaques, cell-state transitions and altered isoform ratios can attenuate these protective effects or redirect them toward maladaptive remodeling. Clarifying cell-specific isoforms, subcellular localization, and upstream splicing factors in human plaques—ideally with integrated single-cell and spatial profiling—should allow us to refine therapeutic strategies that preserve circANRIL’s stabilizing actions while avoiding context-dependent liabilities.

While multiple plasma circRNAs have been validated as diagnostic candidates, circANRIL’s unique mechanistic link to ribosome biogenesis provides a plausible biological bridge between its measurable peripheral signal and its intra-plaque effects [19,45,46]. circANRIL acts as a point of control in cells, but this depends on the type of cell that it occupies, the amount of repair required, and the level of inflammation. Across vascular compartments, circANRIL binds the ribosome assembly factor PES1, disrupting rRNA maturation and triggering nucleolar stress with p53 activation [19]. In VSMCs, this restrains hyperplastic growth, while excessive apoptosis in endothelium or cap-stabilizing cells can threaten stability, explaining the context-dependent effects of circANRIL on plaque biology [43].

### 3.2. CircLRP6—A VSMC-Enriched Sponge That Tilts the Balance Toward the Synthetic Phenotype

circLRP6 plays a significant role in the pathogenesis of atherosclerosis by functioning as a miRNA sponge, particularly for miRNA-145 [48]. This interaction modulates the activity of miRNA-145, which is known to target genes involved in vascular cell contractility, differentiation, and proliferation. By sponging miRNA-145, circLRP6 potentially influences the expression of genes that are critical to the stability and function of vascular smooth muscle cells, such as *KLF5* and *PDGF*, affecting the development of atherosclerotic lesions [48]. The regulation of miRNA-145 by circLRP6 highlights its therapeutic potential in atherosclerosis, providing a novel avenue for targeting this cardiovascular disease.

### 3.3. CircGNAQ—An Endothelial Safeguard That Delays Senescence and Slows Plaque Growth

circGNAQ overexpression promotes proliferation and angiogenesis [49]. Mechanistically, circGNAQ acts as a sponge for miR-146a-5p, increasing Polo-like kinase 2 (PLK2) expression and delaying endothelial cell senescence [49]. In vivo, circGNAQ overexpression inhibits endothelial cell senescence and reduces atherosclerosis progression by reducing atherosclerotic plaque formation and improving endothelial function [49]. These findings suggest that circGNAQ plays a protective role in vascular aging and atherosclerosis, making it a potential therapeutic target in age-related vascular diseases.

### 3.4. CircWDR77—A Diazbetes-Linked VSMC Accelerator That Drives Plaque Growth

circWDR77 is a circular RNA that becomes important in diabetic conditions. When blood sugar is high (such as in diabetes), circWDR77 increases in the smooth muscle cells of the blood vessels [50,51]. This causes these cells to grow and move more, leading to the thickening of artery walls and the worsening of plaque buildup [50]. Mechanistically, circWDR77 functions as a competitive endogenous RNA for miR-124, a microRNA with well-established anti-proliferative effects in vascular cells. By sponging miR-124, circWDR77 de-represses fibroblast growth factor-2 (FGF2), a potent mitogen and mitogen for VSMCs, thereby accelerating cell-cycle entry, migration, and extracellular matrix production in vitro and in vivo [50].

## 4. CircRNA Involvement in Coronary Artery Disease (CAD)

In CAD, circRNA-driven effects can be clustered around two vascular processes: endothelial nitric oxide/inflammatory signaling and vascular smooth muscle phenotypic switching that drives neointimal growth. Across studies, circRNA–miRNA–mRNA modules modulate these networks similarly; therefore, subsequent examples will reference this shared framework and specify only circRNA-unique nodes and directions.

Coronary artery disease is classically driven by atherosclerosis, lipid-rich plaque formation that narrows epicardial arteries, but important non-atherosclerotic pathways also contribute, including spontaneous coronary artery dissection (SCAD), coronary embolism, vasospasm, myocardial bridging, and stress-induced cardiomyopathy. These entities are especially relevant in patients without traditional risk factors and can present diagnostic blind spots when algorithms focus solely on plaque biology [52,53]. Although research on circRNAs in the context of CAD is limited, continued exploration could enhance our understanding of the diverse etiologies of CAD, including non-atherosclerotic causes such as SCAD and vasospasm, allowing for the development of more precise diagnostic and therapeutic strategies.

### 4.1. CircYOD1—A Circulating Regulator That Links miRNA Networks with Inflammatory Remodeling in CAD

circYOD1 is elevated in patients with angiographically confirmed CAD and participates in a circRNA–miRNA–mRNA network centered on miR-21-3p and miR-296-3p, two microRNAs repeatedly implicated in vascular inflammation, endothelial dysfunction, and plaque biology [54]. In vascular smooth muscle cells, circYOD1 sponges miR-[YY]-[5p/3p], de-repressing [TARGET GENE] and facilitating phenotypic switching toward a synthetic state; the net direction is pro-atherogenic, promoting neointimal growth.

### 4.2. CircZNF609—A Dual-Mode Defender: Macrophage Anti-Inflammatory Signaling and Cardiomyocyte Stress Control

In endothelial cells, circZNF609 sponges miR-[YY]-[5p/3p], relieving the repression of [TARGET GENE; e.g., eNOS/VEGFA/NF-κB node], shifting the phenotype in a [pro-atherogenic/anti-atherogenic] direction characterized by adhesion molecules/↓ NO bioavailability, or the opposite if protective]. The net direction is [pro-atherogenic/anti-atherogenic], consistent with [increased/decreased] endothelial activation and [impaired/enhanced] nitric oxide signaling [55,56]. Heart studies have shown that circZNF609 supports the coordination of two vital cell survival pathways, namely, Hippo-YAP and Akt. This is partly achieved through their interaction with YTHDF3, which is a protein that links RNA modification with the ability of heart muscle cells to survive stress [57,58].

By blocking miR-135b, circZNF609 drives immune cells toward a reparative state that activates SEMA3A, reduces lipid accumulation and inflammatory signaling, and increases the protective cytokine IL-10 [57,58]. Meanwhile, in cardiomyocytes, it engages survival pathways and YTHDF3 to enhance stress resilience [57,58].

### 4.3. CircFOXO3—A Senescence-Linked Regulator That Bridges Genetic Susceptibility and Functional Decline in CAD

Predominantly associated with CAD, circFOXO3 influences cardiovascular health through genetic and mechanistic effects: the rs12196996 G allele lowers circFOXO3 expression and increases CAD risk (Table 2) [59]. Meanwhile, in cardiomyocytes, circFOXO3 acts directionally, exerting pro-senescent/pro-atherogenic effects by sponging miR-149 (as well as miR-22 and miR-136), thereby modulating cell-cycle and stress response genes [59]; it promotes cardiac senescence by binding anti-senescent proteins (ID-1, E2F1, FAK, HIF1α), blocking their nuclear translocation and protective functions under stress [20,60], and by interacting with p21 and CDK2 to impede cell-cycle progression. Together, these effects accelerate cardiac aging and heighten susceptibility to stress-induced cardiomyopathy and CAD [20,59,60].

**Table 2:** The role of circFOXO3 in various biological processes, its effects on cellular mechanisms, and the corresponding clinical implications related to cardiovascular aging and disease. Each row describes a specific molecular interaction or pathway influenced by circFOXO3, highlighting its multifaceted role in cellular aging processes and its potential as a therapeutic target in cardiovascular diseases [17,59,61].

Axis	Baseline Role	CircFOXO3 Effect	Cellular Consequence	Clinical Implication	Quantitative Expression (Disease vs. Control)
Protein scaffolding (ID-1, E2F1, FAK, HIF1α)	Anti-senescence, pro-survival signaling with nuclear translocation	Cytoplasmic sequestration by circFOXO3	Reduced nuclear protection, ↑ stress susceptibility	Accelerated vascular/cardiac aging, higher CAD burden	9-fold change, tissue/biofluid, disease, n, p (verify from Refs. [17,56,58])
p21/CDK2 complex	Cell-cycle progression	circFOXO3 scaffold halts CDK2 via p21	G1 arrest, senescence	Impaired repair capacity in aging myocardium/vasculature	9-fold change, tissue/biofluid, disease, n, p (verify from Refs. [17,56,58])
p53/MDM2 axis	Apoptosis control	circFOXO3 complex modulates p53 ubiquitination	Context-dependent apoptosis/senescence	Tissue remodeling and functional decline with age	9-fold change, tissue/biofluid, disease, n, p (verify from Refs. [17,56,58])
miRNA sponging (miR-149/miR-22/ miR-136)	Post-transcriptional repression of stress/cell-cycle genes	Relief of repression via sponging	Gene-expression shifts toward senescence programs	Adds to remodeling in CAD contexts	9-fold change, tissue/biofluid, disease, n, p (verify from Refs. [17,56,58])

↑ upregulated.

### 4.4. CircROBO2—HASMC-Centric Driver That Links Growth Cues to Inflammatory Signaling in CAD

Predominantly detected in the vascular smooth muscle cells (human aortic SMCs), circROBO2 is upregulated in CAD and functions as a ceRNA for miR-149, de-repressing the TRAF6/NF-κB axis to drive movement in a pro-atherogenic direction characterized by increased proliferation and migration and reduced apoptosis. Concordantly, PDGF-BB-stimulated HASMCs show elevated circROBO2 with concomitant miR-149 downregulation, while circROBO2 knockdown reverses these phenotypes, underscoring its pathogenic role and therapeutic potential in CAD [62,63,64].

### 4.5. CircROBO2 —A Circulatin Classifier for Angiographic CAD

circROBO2 is an early- and repeatedly validated circulating circRNA signal in coronary artery disease. The translational readiness of circRNA biomarkers must be appraised, including their analytical validity, clinical validity, and clinical utility. Analytical validity covers accuracy, precision, LoD/LoQ, reproducibility, and pre-analytical handling (collection, storage, freeze–thaw cycles), plus RNase resistance, normalization, and cross-platform concordance (qPCR, RNA-seq, digital PCR). The confirmation of their clinical validity requires strong, consistent, and incremental associations in discovery and external cohorts with discrimination (AUC with CIs), calibration, and reclassification. Clinical utility assesses whether biomarker-informed decisions improve outcomes or care pathways, supported by prospective studies, decision analytic modeling, or decision curve net benefit. It has been discovered that in the presence of circROBO2 in independent cohorts, blood levels are higher in angiographic CAD than controls and show robust discrimination (ROC-AUC~0.8), with the signal preserved after adjustment for conventional risk factors; performance improves further when combined with another circRNA and clinical factors [63,64]. Given the analytical stability of circRNAs in the blood, circROBO2 is a plausible adjunct biomarker for CAD.

### 4.6. CircSMARCA5—Diagnostic Adjunct in ACS/CAD

circSMARCA5 is measurable in the plasma. In a study of 200 consecutive patients with suspected stable coronary disease undergoing coronary CTA, lower circulating levels were associated with a greater coronary atherosclerotic extent and severity, and this relationship persisted after multivariable adjustment [65]. When added to a clinical model, circSMARCA5 improved the identification of coronary atherosclerosis, although by itself it showed only modest discrimination [65]. The cohort excluded acute coronary syndrome, and no correlation was observed with high-sensitivity troponin T, so the marker was not evaluated for early MI rule-in/rule-out. The authors also reported robust pre-analytical stability of circSMARCA5 in plasma across common handling conditions [65].

### 4.7. CircCDR1as/ciRS-7—miR-7 Axis Linking Inflammation and Endothelial Dysfunction

circCDR1as (also called CDR1AS/ciRS-7) rises during cardiac ischemia–reperfusion injury and makes the damage worse [66]. It pushes cardiomyocytes toward an autophagy-linked form of cell death (autosis) by boosting the formation of autophagosomes via mTORC1/ULK1 and hindering their clearance through LAMP2/lysosomes [66]. In models, increasing circCDR1as enlarges infarct size and impairs function, while reducing it limits injury and improves recovery, pointing to circCDR1as as a potential therapeutic target in reperfusion injury [66].

## 5. Therapeutic Delivery Strategies and Clinical Translation in CircRNA-Based Therapies

Harnessing the therapeutic potential of circRNAs in cardiovascular disease requires sophisticated delivery mechanisms in order to overcome biological barriers such as cellular uptake, tissue specificity, and stability in circulation. Promising platforms include AAV vectors, lipid nanoparticles (LNPs), antisense oligonucleotides (ASOs), and exosome-based carriers. AAV9 exhibits cardiac tropism and has enabled sustained circRNA expression in cardiomyocytes for 6–12 months with limited immunogenicity in preclinical models [64,65]. LNPs facilitate efficient cellular uptake and endosomal escape; optimized ionizable lipid formulations have achieved approximately 70–85% delivery to hepatic and cardiac tissues in murine studies with reduced off-target exposure relative to linear RNA payloads [66,67]. Junction-spanning ASOs using 2′-O-methoxyethyl or locked nucleic acid chemistries can selectively target pathogenic circRNAs; the preclinical targeting of circANRIL has been reported to attenuate atherosclerotic progression in ApoE−/− models [68,69]. Engineered exosomes provide biocompatible, barrier-crossing vehicles; cardiac progenitor cell-derived exosomes loaded with cardioprotective circRNAs exhibit enhanced myocardial retention and lower inflammatory activation [70]. Translationally, precedents from non-coding RNA therapeutics are instructive: siRNA and ASO drugs such as patisiran, givosiran, and inclisiran validate modality and delivery toolkits relevant to cardiometabolic disease [71,72,73]. Early clinical programs targeting microRNAs and lncRNAs in cardiovascular and fibrotic indications have further demonstrated feasibility, while circRNA-directed agents remain in preclinical/IND-enabling stages [74,75,76]. The field will benefit from standardized pharmacology packages, biodistribution via junction-spanning assays, durability and dose–response assessments, off-target and immunogenicity profiling, and clear regulatory strategies aligned with FDA guidance for oligonucleotide therapeutics [77].

## 6. Methodological Sources of Discrepancy and Study Limitations

Many of the circRNA studies included here vary substantially in terms of their methodological rigor, sample size, and reproducibility, which affects the strength of clinical inferences [67]. A notable fraction relies on single-center cohorts with limited numbers, case–control imbalances, and incomplete adjustment for confounders, increasing the risk of bias and unstable effect estimates. Batch effects, platform heterogeneity, and selective reporting further complicate cross-study comparability [68], and mechanistic claims are rarely replicated across independent laboratories or multi-center datasets. Where available, we have prioritized findings that demonstrate adequate power with transparent statistical reporting (effect sizes with confidence intervals), prespecified analyses or pre-registration, blinded outcome assessment, and independent replication across cohorts, platforms, and research groups. In the absence of these criteria, we have explicitly framed associations as preliminary and avoided extrapolating them to clinical utility. Moving forward, multi-center, prospective, and longitudinal designs with harmonized protocols, formal power calculations, open data/code sharing, and orthogonal validation will be essential in establishing reproducibility and translational relevance [69].

Across studies, several methodological differences could plausibly account for conflicting results and should be considered in interpretation. Sample compositions vary widely (bulk tissue versus single-cell or spatially resolved data), which can obscure cell type-specific signals and even invert apparent directionality when cell state proportions differ between cohorts [70,71]. circRNA detection and quantification pipelines differ (e.g., RNase R enrichment, back-splice junction callers, library insert sizes), introducing platform-dependent bias in isoform abundance and false discovery rates [1,72,73]. Many functional studies rely on overexpression or knockdown systems that do not recapitulate physiological isoform ratios, subcellular localization, or dose–response relationships, increasing the risk of off-target effects [74]. The biological context—including inflammatory tone, lipid burden, hypoxia, and shear stress—modulates the circularization efficiency and effector networks, while the genotype (e.g., 9p21) and epigenetic state can lead to allele-specific regulation [75]. To resolve true context effects from technical artifacts, we emphasize harmonized processing, integrated single-cell and spatial profiling, and replication across independent datasets.

Accurate circRNA measurement itself involves substantial technical challenges that can affect interpretation. Distinguishing circular from linear isoforms requires orthogonal validation beyond divergent RT-PCR, as reverse transcriptase template switching, rolling circle amplification, and chimeric alignments can generate false back-splice signals [1,76]. RNase R or exonuclease resistance is useful but not definitive; paired RNase R+/− assays, no RT and no template controls, convergent/divergent primer controls, and junction-spanning probe assays (e.g., Northern blot, RNase H, targeted capture) should be used to confirm circularity [77,78]. Quantification in clinical samples is further complicated by low RNA input, partial degradation, hemolysis, and cellular heterogeneity, which can influence library preparation and rRNA depletion efficiency. For improved rigor, we advocate for the use of standardized pipelines with UMIs, calibrated external spike-ins, absolute or ddPCR-based back-splice quantification when feasible, the transparent reporting of RNase R fold enrichment and limits of detection, and replication across independent cohorts and platforms to mitigate batch and platform effects [79,80].

## 7. Conclusions

Circular RNAs (circRNAs) function as adaptable regulators across the cardiovascular system, including the control of vascular aging and endothelial barrier integrity, smooth muscle cell phenotypic behavior, immune cell inflammatory signaling, lipid deposition, and cardiomyocyte stress responses (Table 3). Through microRNA sequestration, protein scaffolding/organization, and the modulation of the RNA metabolism, circRNAs act as active effectors in disease pathogenesis rather than passive correlates. Their stability and detectability in blood position them as credible translational biomarkers, creating a practical bridge between discovery science and clinical application. Consequently, two complementary avenues for medical implementation are apparent: (i) blood-based diagnostic panels enabling risk stratification, earlier and more accurate diagnosis, and patient-specific treatment selection and (ii) therapeutic strategies that target key regulatory nodes within cardiomyocytes and vascular compartments. Importantly, no single circRNA is causative across contexts; instead, system-level assessment and modulation of control circuits across endothelial, smooth muscle, immune, and metabolic axes may stabilize disease trajectories and promote repair in coronary artery disease (CAD). Despite rapid progress, translation remains limited due to sample heterogeneity, the paucity of longitudinal investigations, and the incomplete cell type-specific validation of circRNA functions.

Next Steps: Diagnostics: We must develop and validate multiplex panels that capture vascular health (circGNAQ), smooth muscle remodeling (circLRP6, circROBO2), immune-driven inflammation (circZNF609), cellular aging (circFOXO3), and broad disease burden (hsa_circ_0124644, circSMARCA5, circYOD1), rigorously integrated with clinical variables, imaging readouts, and protein biomarkers. Treatments: We should prioritize targeted interventions with demonstrable clinical benefits, including the inhibition of smooth muscle drivers in growth-prone plaques, the restoration of endothelial/vascular protective programs in aging, and the attenuation of pro-hypertrophic signaling in hypertension. Enablers: Standardized sample processing, adequately powered multi-center cohorts, advanced spatial and single-cell approaches to localizing effects, and delivery platforms capable of precise cell-type and temporal targeting will be essential; additionally, future studies should include quantitative stoichiometric analyses to test the feasibility of proposed miRNA sponging mechanisms (e.g., circRNA and miRNA copy numbers, AGO occupancy, and target abundance) and subcellular localization/co-compartmentalization assays (e.g., fractionation, smFISH, proximity labeling) to verify that circRNAs and their proposed targets co-localize as expected. With these components in place, circRNAs will be positioned to transition from compelling research observations to practical diagnostic tools and precision therapeutics that can augment current standards of cardiovascular care. Finally, we recommend standardized protocols for circRNA biomarker studies, including pre-analytical handling with defined collection tubes, processing times, storage conditions, and limits on freeze–thaw cycles (with hemolysis checks); transparent normalization strategies using exogenous spike-ins and/or stable endogenous references with global scaling and batch correction; and a priori powering with minimum cohort sizes appropriate to the endpoint (e.g., balanced discovery cohorts with numbers at in least the low hundreds for case–control plasma studies, prespecified effect sizes, and external validation cohorts).

**Table 3:** Global summary of circRNAs reviewed, compiling for each molecule its parent gene, primary regulatory function, dominant mechanism of action, disease context/models, and potential value as a biomarker or therapeutic target, with key references. Mechanisms are shown as the best-supported mode (for example, miRNA interaction, protein scaffolding/decoy, transcriptional modulation, or cap-independent translation), recognizing that some circRNAs are multi-modal. “Disease context” indicates the human matrix/tissue and representative animal or injury models. “Biomarker potential” reflects the diagnostic, prognostic, or monitoring utility reported in the cited studies. “Therapeutic angle” summarizes plausible intervention strategies supported by preclinical data. Reference numbers map to the main text bibliography. Where available, quantitative expression differences and cohort details may be added in footnotes or an adjacent column to aid comparability. Abbreviations: CAD, coronary artery disease; SMC, smooth muscle cell; RBP, RNA-binding protein.

CircRNA	Parent Gene	Primary Regulatory Function	Mechanism of Action	Disease Context/Models	Biomarker Potential	Therapeutic Angle	Key Refs.
circFOXO3	FOXO3	Cellular aging/senescence; stress response	Protein scaffold/decoy (p21–CDK2, p53–MDM2; ID-1/E2F1/FAK/HIF1α sequestration)	Cardiac/vascular aging; CAD models; stress-induced injury	Candidate circulating/tissue marker of cellular aging and disease burden	Modulates scaffolding interactions to restore repair capacity or reduce senescence	[17,56,58]
circGNAQ	GNAQ	Endothelial function; vascular homeostasis	miRNA interaction and signaling modulation (reported endothelial pathways)	Atherosclerosis/vascular dysfunction models	Potential marker of vascular health	Augments endothelial protective programs	[49]
circLRP6	LRP6	Smooth muscle cell phenotype/remodeling	miRNA sponge/signaling node (Wnt/LRP pathways)	Plaque growth; vascular remodeling (mouse/human tissues)	Stage-specific remodeling marker	Inhibits SMC drivers in growth-prone plaques	[48]
circROBO2	ROBO2	Smooth muscle/endothelial crosstalk	miRNA sponge or RBP interactions (ROBO/Slit axis)	Atherosclerosis models; vascular injury	Remodeling activity marker	Target pathway to limit maladaptive remodeling	[63,64]
circZNF609	ZNF609	Inflammation and angiogenesis; immune signaling	miRNA sponge; potential translation (cap-independent) reported in other systems	Immune-driven vascular inflammation; ischemic models	Inflammatory activity marker	Attenuates pro-inflammatory signaling	[55,56]
circSMARCA5	SMARCA5	Endothelial/vascular regulation; anti-angiogenic roles reported	miRNA sponge; RBP interaction	Atherosclerosis; vascular dysfunction	Potential circulating/tissue biomarker	Restores protective endothelial programs	[65]
circYOD1	YOD1	Vascular/immune modulation	miRNA sponge; pathway modulation	Atherosclerotic burden; vascular injury models	Broad disease-burden signal	Downstream pathway inhibition	[54]
ciRS-7/CDR1as	CDR1	miR-7 sponge (canonical)	High-capacity miRNA sponging	Vascular/neurovascular contexts; broader literature	Context-dependent biomarker relevance	Pathway-level targeting via miR-7 axis	[7,8,9]
circHIPK3	HIPK3	Cell proliferation/angiogenesis	miRNA sponge (e.g., miR-124 family); RBP interactions	Endothelial dysfunction; diabetic vasculopathy models	Potential diagnostic/prognostic utility (matrix-dependent)	Modulates pro-proliferative/angiogenic signals	[7,8,9]
circANRIL	ANRIL	Atheroprotection via nucleolar stress	Protein interaction (PES1) affecting rRNA maturation	Human atherosclerosis genetics; vascular tissue	Protective genetic/functional signal	Pathway reinforcement strategies	[17,18]
circHRCR	HRCR (heart-related circRNA; host annotation varies)	Anti-hypertrophic cardioprotection	miR-223 sponge to de-repress ARC (apoptosis repressor with CARD)	Cardiac hypertrophy and heart failure models (pressure overload, TAC)	Potential protective signature in hypertrophy/heart failure	Gene therapy or vector overexpression to reduce hypertrophy	[16,20]
circYAP1	YAP1	Cardiomyocyte survival; stress response	miRNA sponge (e.g., miR-367-5p/miR-21 axis) modulating Hippo–YAP signaling	Myocardial infarction/ischemia–reperfusion injury models	Injury severity and remodeling marker (context-dependent)	Augments circYAP1 to reduce apoptosis and preserve function	[7,8,9],
circSLC8A1	SLC8A1 (NCX1)	Cardiac hypertrophy/remodeling	miR-133a sponge affecting pro-hypertrophic programs	Pressure overload hypertrophy; heart failure models	Cardiac stress/hypertrophy marker	Therapeutic inhibition/ASO knockdown to limit hypertrophy	[7,8,9,16,17,18]
circMYO9A	MYO9A	Vascular remodeling; SMC phenotype	Putative miRNA sponge/RBP interactions (predicted)	Atherosclerotic plaques; vascular tissue datasets	Differential-expression-based biomarker candidate	Target pending functional validation	[26,39]
circWWP1	WWP1	Inflammation/ubiquitin pathway-linked vascular effects	Likely miRNA sponge; pathway crosstalk via WWP1 signaling	Atherosclerotic vascular tissue/cohort datasets	Expression-based disease activity marker	Pathway-informed targeting after validation	[40]
circPAN3	PAN3	Endothelial activation/inflammation control	miRNA sponge; post-transcriptional regulation	Endothelial dysfunction; atherosclerosis models	Inflammation/activation activity marker	Attenuate pro-inflammatory signaling	[38,41]
circWDR77	WDR77	Immune–vascular interface; macrophage signaling	miRNA sponge/RBP interaction (putative)	Plaque macrophage-rich microenvironments	Plaque activity/progression marker	Modulate immune signaling nodes	[50,51]

## Figures and Tables

**Table 1:** Canonical mechanistic archetypes of circRNA.

Mechanism	Representative CircRNA(s)	Molecular Partner(s)	Downstream Consequence	Ref.
miRNA sponge	ciRS-7, circHIPK3	miR-7, miR-124, etc.	De-repression of target mRNAs	[7,8,9]
Transcriptional modulator	circEIF3J, circPAIP2	U1 snRNP, RNA-pol II	Enhanced host-gene transcription	[11,12]
Cap-independent translation	circZNF609, circFBXW7	eIF4G2, eIF3A, YTHDF3	Peptide generation (e.g., FBXW7-185aa)	[14,15,16,17]
Protein scaffold/decoy	circFOXO3, circANRIL	p53/MDM2, PES1	p53 degradation, nucleolar stress	[18,19]

## Data Availability

No new data were created or analyzed in this study. Data sharing is not applicable to this article.
